# Understanding disruption of the gut barrier during inflammation: Should we abandon traditional epithelial cell lines and switch to intestinal organoids?

**DOI:** 10.3389/fimmu.2023.1108289

**Published:** 2023-02-16

**Authors:** Susana Lechuga, Manuel B. Braga-Neto, Nayden G. Naydenov, Florian Rieder, Andrei I. Ivanov

**Affiliations:** ^1^ Department of Inflammation and Immunity, Lerner Research Institute, Cleveland Clinic Foundation, Cleveland, OH, United States; ^2^ Department of Gastroenterology, Hepatology and Nutrition, Digestive Diseases and Surgery Institute, Cleveland Clinic Foundation, Cleveland, OH, United States

**Keywords:** actin cytoskeleton, adherens junctions, colonoids, cytokines, enteroids, epithelial barrier, inflammatory bowel diseases, tight junctions

## Abstract

Disruption of the intestinal epithelial barrier is a hallmark of mucosal inflammation. It increases exposure of the immune system to luminal microbes, triggering a perpetuating inflammatory response. For several decades, the inflammatory stimuli-induced breakdown of the human gut barrier was studied *in vitro* by using colon cancer derived epithelial cell lines. While providing a wealth of important data, these cell lines do not completely mimic the morphology and function of normal human intestinal epithelial cells (IEC) due to cancer-related chromosomal abnormalities and oncogenic mutations. The development of human intestinal organoids provided a physiologically-relevant experimental platform to study homeostatic regulation and disease-dependent dysfunctions of the intestinal epithelial barrier. There is need to align and integrate the emerging data obtained with intestinal organoids and classical studies that utilized colon cancer cell lines. This review discusses the utilization of human intestinal organoids to dissect the roles and mechanisms of gut barrier disruption during mucosal inflammation. We summarize available data generated with two major types of organoids derived from either intestinal crypts or induced pluripotent stem cells and compare them to the results of earlier studies with conventional cell lines. We identify research areas where the complementary use of colon cancer-derived cell lines and organoids advance our understanding of epithelial barrier dysfunctions in the inflamed gut and identify unique questions that could be addressed only by using the intestinal organoid platforms.

## Introduction

**Figure 1:**
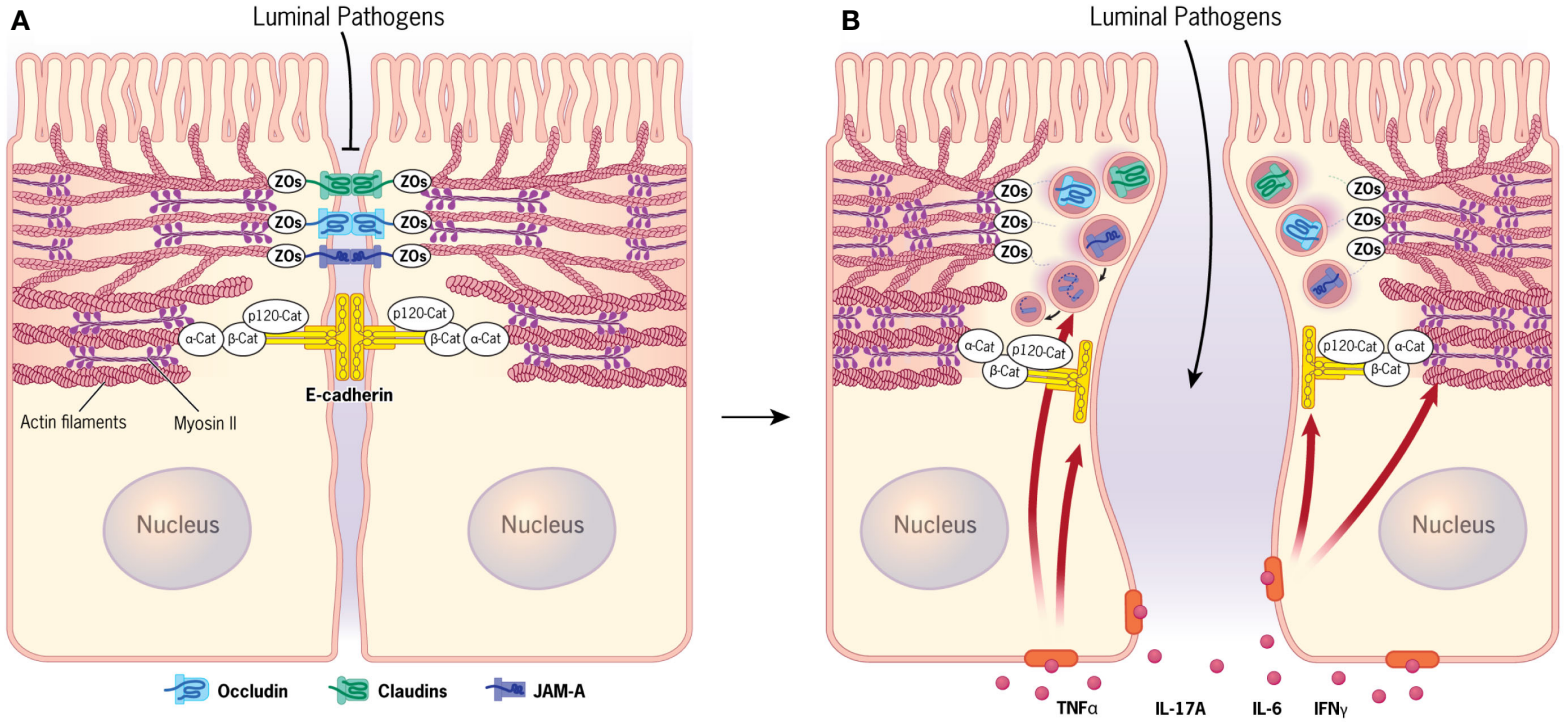
Apical junctions in normal and inflamed intestinal epithelium. **(A)** Normal intestinal epithelial barrier is established by assembly of tight junctions and adherens junctions. Both junctional complexes are physically associated with the circumferential cytoskeletal belt containing actin bundles and myosin II filaments. **(B)** Signaling by different cytokines accumulating in the inflamed intestinal mucosa triggers disruption of the epithelial barrier that is driven by removal of junctional proteins from the intercellular contacts and remodeling of the perijunctional actomyosin cytoskeleton. **Figure 1** by Gwendolyn Fuller, MFA. Reprinted with the permission of the Cleveland Clinic Center for Medical Art & Photography ^©^ 2023. All Rights Reserved.

The intestinal epithelium plays crucial homeostatic roles by forming the protective barrier that separates the internal organs from the luminal microbiota and regulates bidirectional fluxes of water, solutes, nutrients, and waste. These functions are enabled by structural adaptations of intestinal epithelial cells (IEC) that include extensive intercellular contacts and the apico-basal cell polarity (1, 2). Both the intercellular contacts and the polarized architecture of IEC are mediated by elaborate epithelial junctions (3, 4). The two most apically located junctional complexes, tight junctions (TJs) and adherens junctions (AJs), are known to be critical for controlling permeability of the gut barrier, whereas the role of the third junctional complex, desmosomes, remain less understood (3, 4). TJs and AJs represent multiprotein platforms assembled at the plasma membrane that contain transmembrane adhesive and cytoplasmic scaffolding proteins (**Figure 1A**) (3–5). Transmembrane components of TJs, such as claudins, occludin, and junctional adhesion molecule A (JAM-A), form adhesive bonds with their partners on the opposing cell membrane, whereas on the cytoplasmic side of the membrane, they interact with different scaffolds, most notably members of a ‘*zonula occludens*’ (ZO) protein family (5, 6). A predominant transmembrane AJ protein, E-cadherin, participates in homotypic interactions with other E-cadherin molecules at the cell surface and makes complexes with cytoplasmic β-catenin, p120-catenin, and α-catenin proteins (7–9).

Proper plasma membrane assembly of different TJ and AJ components is necessary, but not sufficient, to create and maintain the functional intestinal epithelial barrier. An additional mechanism critical for barrier regulation involves the coupling of epithelial junctions to the underlying cortical actin cytoskeleton. A circumferential actin filament belt enriched with an actin motor, non-muscle myosin II (NM II), is a prominent structural feature of well-differentiated IEC (**Figure 1A**) (10–15). This perijunctional actomyosin belt generates mechanical forces, which stabilize and remodel both AJs and TJs. It also transduces and integrates signaling from different extracellular stimuli and intracellular molecular pathways to modulate tightness of the gut barrier under homeostatic and disease states (10–15).

Disruption of the intestinal epithelial barrier is a well-recognized hallmark of mucosal inflammation. This phenomenon is best studied in chronic autoimmune disorders, such as inflammatory bowel diseases that include Crohn’s disease (CD) and ulcerative colitis (UC), as well as celiac disease (16–20). Additionally, gut barrier leakiness appears to be a common feature for many extraintestinal and systemic disorders with inflammatory etiology, such as asthma, sepsis, diabetes, multiple sclerosis, etc. (21–23). It should be noted that increased permeability of IEC barrier in CD and UC patients develops in parallel to other defects of the intestinal host defense. Such defects can include dysfunctions of Paneth cells leading to diminished secretion of antimicrobial peptides (24–27) and also abnormal mucin production by Goblet cells (24, 28–30). The combined impairment of the cellular and secretory epithelial defense mechanisms in the gut result in excessive exposure to luminal microbiota, leading to activation of the immune response and release of various inflammatory mediators in the intestinal mucosa (17, 19, 21, 23, 31). These inflammatory mediators, including tumor necrosis factor (TNF)-α, interleukins, and interferon (IFN)-γ, act on IEC to accelerate TJ/AJ disassembly and barrier leakiness, thereby accelerating mucosal inflammation (**Figure 1B**) (31–35).

Multiple mechanisms could contribute to the junctional disassembly and disruption of the epithelial barrier in the inflamed intestinal mucosa (17–19, 36). These mechanisms include the decreased expression of different AJ and TJ proteins, dysregulated vesicular trafficking of junctional components, as well as altered assembly and contractility of junction-associated actomyosin cytoskeleton (17, 18, 36, 37). Such complexity of molecular triggers and mechanisms requires careful selection of appropriate *in vitro* models to recapitulate and dissect the signaling and cellular responses characteristic of the inflamed human intestine *in vivo*. Until recently, the experimental toolbox for *in vitro* studies of the human intestinal epithelial barrier included just a handful of well-differentiated colon cancer-derived cell lines (38). While studies of these model cell lines provided a crucial foundation for our understanding of the structure and function of IEC junctions, whether obtained data faithfully reflect the dynamics and regulation of the gut barrier *in vivo* is a lingering question.

Development of a new technology allowing *ex vivo* growth and differentiation of primary intestinal organoids provided a major methodological breakthrough in investigations of gastrointestinal physiology and diseases. This technology has been increasingly applied to study structure and regulation of the intestinal epithelial barrier, resulting in the accumulation of a new wealth of important experimental data while also revealing some limitations of this methodology. It is necessary, therefore, to understand how well the newest data obtained with primary intestinal organoids align with older studies that utilized conventional colonic epithelial cell lines and, in addition, what unique aspects of gut barrier regulation during intestinal inflammation could be discovered using primary human organoids. This review attempts to address these important questions. In order to provide a logical in-depth description of the data, we will focus on human intestinal organoids and colonic epithelial cell lines and minimizing discussions of the data obtained using rodent models. Furthermore, the attention will be limited to responses of primary organoid and conventional IEC to the most studied inflammatory cytokines and some bacterial compounds, such as lipopolysaccharide (LPS), but will exclude studies of intestinal epithelial interactions with bacteria and viruses. Such a rapidly expanding field is largely focused on examining the inflammatory responses and has been recently summarized in several excellent reviews (39–41).

## Conventional cell culture models for *in vitro* studies of human intestinal epithelial barrier

The vast majority of studies that model disruption of the intestinal epithelial barrier during mucosal inflammation *in vitro* have been performed using a limited set of colonic epithelial cells lines that include T84, Caco-2, and HT-29 cells. All these cell lines originate from human colon carcinoma samples. T84 cells were derived from lung metastasis, and this cell line was established after serial passaging of the tumor specimens in nude mice (42). Caco-2 and HT-29 cells were derived from primary colon tumors (43). When growing on permeable membrane support, T84 cells form well-polarized cell monolayers with developed junctional complexes and a tight paracellular barrier (44–49). Caco-2 cell monolayers also assemble robust apical junctions and establish the paracellular barrier, although not as tight as developed by T84 monolayers (50). Both parental Caco-2 cells and its more differentiated clone, Caco-2BBE, have been used to study IEC junctions (51–54). Unlike T84 and Caco-2 cells, the parental HT-29 cell line is poorly differentiated, and these cells do not form a tight paracellular barrier. However, several well-differentiated clones of HT-29 cells, most notably HT-29/B6 (55–57) and HT-29cf8 (58–60) have been used to study barrier properties and the molecular organization of IEC junctions.

Despite the fact that T84, Caco-2 cells, and HT-29 clones have the morphological characteristics of mature IEC, establish the epithelial barrier, and respond to inflammatory cytokines with barrier disruption, there are several reasons why these cell lines cannot faithfully recapitulate the structural/molecular features and regulation of a normal intestinal epithelium. First, the colonic cancer cell lines have multiple karyotypic abnormalities that include chromosome loss and amplification (61). Consistently, a whole genome transcriptomic and proteomic analyses observes large differences in gene and protein expression profiles between all three model IEC lines and a normal human intestinal epithelium (62, 63). Second, T84, Caco-2, and HT-29 cells possess several oncogenic mutations that could affect junctional integrity and inflammatory signaling. Most common are mutations of the tumor suppressors, p53 and adenomatous polyposis coli (APC), as well as mutations of KRAS, PIK3CA, BRAF, and CTNNB1 genes (64–68). APC and CTNNB1 mutations could directly affect the structure and adhesive properties of AJs by modulating the junctional recruitment of β-catenin (69, 70). Mutations of p53, KRAS, PIK3CA, and BRAF could have indirect effects on the IEC barrier and apical junctions by altering the architecture and dynamics of the perijunctional actin cytoskeleton (71–74). Likewise, extensive pro-oncogenic changes in the genetic landscape of colon cancer cell lines could affect their responses to various inflammatory stimuli, thereby adding concerns about utilizing these cell lines to model molecular pathway characteristics for the inflamed intestinal mucosa *in vivo*. Finally, well-differentiated T84, Caco-2, and HT-29 monolayers are composed of a relatively homogenous cell population resembling colonic enterocytes and are devoid of the complexity of the gut epithelium that also contains other cellular types, such as Goblet, Paneth, and enteroendocrine cells.

## Development of primary human intestinal organoids and measuring integrity of their epithelial barrier

Intestinal organoids represent *ex vivo* self-organizing cellular structures that mimic the complex organization of gut tissue. Development of the organoids depends on the establishment and maintenance of the intestinal stem cell ‘niche’ with stem cell differentiation into various gut-like structures depending on the chemical composition and mechanical properties of the environment (75). Initially established with mouse small intestinal organoids (76), this technology has been successfully applied to mimic different segments of the human gastrointestinal system (77). Organoid cultures derived from the small intestine are referred to as ‘enteroids’, whereas colon-derived structures are called ‘colonoids’ (78). A comprehensive description of organoid development, features, and applications is provided by several excellent recent reviews (75, 79–82) and will not be detailed here. We will briefly describe major types of human intestinal organoids and outline methodological approaches commonly used to measure epithelial barrier permeability in these model systems.

**Figure 2:**
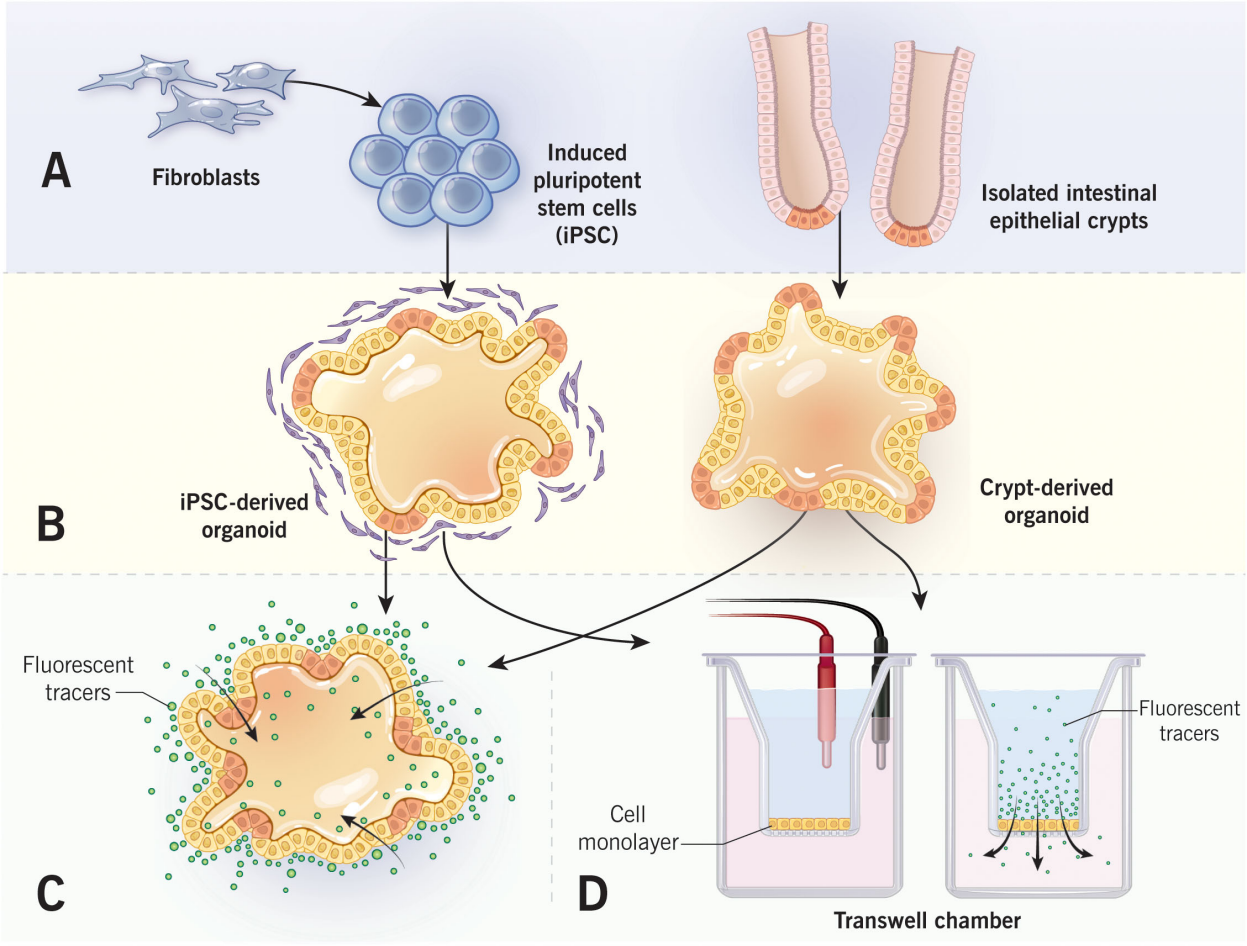
Measurements of epithelial barrier permeability in human intestinal organoids. Intestinal organoids that are derived from either induced pluripotent stem cells or isolated intestinal crypts **(A)** form cyst-like structure after embedding into 3-D extracellular matrix **(B)**. Epithelial permeability could be evaluated in a 3-D spherical organoids by monitoring the passage of fluorescent markers into organoid lumen **(C)**. Alternatively, organoids could be cultured as 2-D epithelial monolayers in Transwell chambers **(D)**. Permeability of organoid-derived monolayers could be examined by measuring either transepithelial electrical resistance, or transmonolayer flux of fluorescent markers. **Figure 2** by Gwendolyn Fuller, MFA. Reprinted with the permission of the Cleveland Clinic Center for Medical Art & Photography ^©^ 2022. All Rights Reserved.

Human intestinal organoids could be generated from either somatic or pluripotent stem cells (**Figure 2**). Somatic cell-derived organoids are driven by adult stem cells that populate isolated small intestinal or colonic crypts. The isolated crypts are mounted into the extracellular matrix (ECM) scaffold, such as Matrigel, and supplied with stem cell niche factors that for human organoids include R-spondin, Noggin, EGF, and Wnt3a (75, 80). ECM-embedded organoids initially form spherical cyst-like structures with central lumen and depending on the environment could acquire more complex morphology, including crypt-like protrusions in small intestinal enteroids (**Figure 2**). The organoids could be propagated and studied in the 3-D culture or dissociated and plated on permeable membrane supports where they form polarized 2-D monolayers (79, 83). The adult stem cell-derived organoids more closely recapitulate the intestinal epithelial layer *in vivo* since they do not possess either chromosomal abnormalities or oncogenic mutations typical for colon cancer cells. Furthermore, they contain different IEC lineages, which proportions could be experimentally modulated by altering stem cell niche factor signaling (84). This attractive experimental system, however, has its own limitations. One is the lack of the mesenchymal/stromal compartment and immune cells, which precludes studying the regulatory cross-talks between IEC and other mucosal/submucosal cell types. Loss of intestinal intraepithelial lymphocytes (IEL) in crypt derived organoids can be considered as a particular shortcoming of this experimental system. IEL, especially TCRγδ and TCRαβ T cells, are emerging regulators of intestinal homeostasis, host-microbiota interactions and mucosal inflammation (85, 86). Importantly, IEL directly interact with different IEC lineages and regulate barrier integrity, repair, and host antimicrobial defenses (87–89). Other limitations of adult stem cell-derived intestinal organoids are the epigenetic and phenotypic changes that may occur following multiple passages ex vivo, including DNA methylation and cell senescence (90, 91). Finally, there is an issue with data reproducibility since organoids are currently derived from a limited number of different individuals and samples obtained from varying locations within the gut. Future comprehensive and better documented studies of human intestinal organoids with large numbers of individuals are required to separate meaningful population data variability from the experimental noise.

An alternative approach involves generating intestinal organoids from human pluripotent stem cells (PSC). The PSC could be established from either pluripotent embryonic stem cells or by inducing differentiated cells, such as fibroblasts, to convert into the pluripotent stage (92, 93). Human intestinal organoids could be generated from PSC *via* a multistep process involving initial stem cell conversion into appropriate germ layers with subsequent differentiation into tissue-specific organoids (92, 94). Importantly, organoids resembling either the small intestine or colon could be generated by manipulating bone morphogenic protein signaling (92, 94). Unlike somatic stem cell-derived intestinal organoids that contain only epithelial cells, PSC-derived organoids contain both epithelial and mesenchymal compartments, thereby better representing the complexity of the gut tissue. A major drawback for this experimental system, however, is that generation of PSC-derived organoids is a complex multistep process that requires significant expertise and resources. Furthermore, PSC-derived organoids do not differentiate *in vitro* and need to be transplanted in the kidney capsule of immunocompromised mice in order to achieve complete differentiation (94). As any labor-intensive sophisticated technology generation of PSC-derived organoids is difficult to standardize, and data reproducibility becomes a problem.

Differentiated intestinal organoids provide a unique opportunity to study the assembly and regulation of the gut epithelial barrier either in heterogeneous populations of primary epithelial cells or under more complex conditions involving epithelial-stromal interactions. Different experimental approaches have been developed to measure barrier permeability at two distinct morphological states of the organoids (3-D spheroids versus 2-D monolayers) (38). Permeability of 3-D organoids has been evaluated by measuring transepithelial flux of marker molecules between the organoid lumen and the environment (**Figure 2**). Two commonly used permeability markers are FITC-Dextran (4,000 Da) and a much smaller fluorescent dye, Lucifer Yellow (95–98). These markers are most frequently added to the cell culture medium, and their accumulation in the organoid’s lumen is measured by fluorescence microscopy (95, 97, 98). Alternatively, the fluorescent marker could be injected into the organoid lumen with subsequent measuring of the decrease in its luminal fluorescence intensity over time (96, 99). When intestinal organoids are cultured as 2-D monolayers on permeable membrane supports, their barrier permeability could be measured using the same experimental approaches developed for conventional epithelial cell monolayers (38, 100). Specifically, transepithelial electrical resistance (TEER) and apical-to-basal flux of FITC dextran or other fluorescent markers are used to measure paracellular ionic permeability and the passage of large uncharged molecules, respectively (**Figure 2**) (38, 100). Finally, junctional structure could be studied in both 2-D and 3-D organoids by using either transmission electron microscopy or by immunolabeling and confocal microscopy of different TJ and AJ proteins.

## Barrier-disrupting effects of inflammatory mediators in human intestinal organoids and conventional IEC lines

*Intestinal crypt-derived organoids*. Since the introduction of primary human intestinal organoids, they have been increasingly used for understanding the effects of inflammatory cytokines on barrier properties as well as the structure of epithelial junctions either in 3-D spheroids or 2-D organoid-derived monolayers. Most studies focused on the effects of TNFα and IFNγ because these cytokines are essential for IBD pathogenesis and have been extensively investigated using conventional human IEC lines (**Table 1**) (11, 31–33, 36).

**Table 1:** Effects of inflammatory mediators on barrier properties of human intestinal organoids and conventional IEC lines.

Inflammatory stimulus	Concentration	Model system	Specific set up	Cell lineages present	Effects on barrier and junctions	Reference
TNFα	4-50 ng/mL	Organoid	Normal enteroid monolayers	E, G, IS	Increased permeability; increased TJ tortuosity.	(101)
TNFα	30 ng/mL	Organoid	Normal 3-D enteroids	E, G	Decreased ZO-2 and ZO-3 expression; increased MLCK expression and Claudin-2 expression	(102)
TNFα	30 ng/mL	Organoid	iPSC derived 3-D organoids	E, EE, G, M, P	Increased permeability; disruption of the TJ structure	(103)
TNFα	10-100 ng/mL	Cell line	Caco-2	E	Increased permeability; decreased ZO-1 expression; increased TJ tortuosity	(104, 105)
TNFα	10 ng/mL	Cell line	Caco-2	E	Increased MLCK expression and activity	(106)
TNFα	100 ng/mL	Cell line	Caco-2	E	Decreased E-cadherin, ZO-1 and occludin expression	(107)
IFNγ	50 ng/mL	Organoid	Normal 3-D colonoids	NR	Increased permeability; no effect on ZO-1 and occludin levels	(108)
IFNγ	200 ng/mL	Organoid	Normal 3-D colonoids	NR	Increased permeability; decreased ZO-1 and occludin levels	(99)
IFNγ	100 ng/ml	Organoid	CD 3-D colonoids	E, EE, G, IS	Increased permeability	(109)
IFNγ	10 ng/mL	Organoid	iPSC derived 3-D organoids	NR	No effects on the AJ or TJ integrity; decreased Claudin-15 expression	(110)
IFNγ	50 ng/mL	Cell line	T84	E	Increased permeability; no effect of ZO-1 and occludin levels	(108)
IFNγ	100 U/mL	Cell line	T84	E	Increased permeability; selective TJ disassembly; no effect of ZO-1 and occludin levels	(44, 111)
IFNγ	10-100 ng/mL	Cell line	T84	E	Increased permeability; decreased ZO-1 and occludin expression	(112, 113)
IFNγ + TNFα	10 ng/mL each	Organoid	iPSC derived monolayers	E, EE, G, IS, P	Increased permeability; disruption of E-cadherin and ZO-1 localization	(114, 115)
IFNγ + TNFα	100 U/mL	Cell line	T84	E	Increased permeability; selective TJ disassembly	(44)
IFNγ + TNFα	10 ng/mL + 2.5 ng/mL	Cell line	Caco-2	E	Increased permeability; selective TJ disassembly	(116, 117)
IFNγ + TNFα + IL-1β	20 ng/mL each	Organoid	CD 3-D colonoids	NR	Increased permeability; decreased claudin-1, angulin-1, E-cadherin and β-catenin expression; increased claudin-2 level	(118)
IL-6	10 ng/mL	Organoid	Normal 3-D colonoids	NR	Decreased occludin and claudin-1 expression	(119)
IL-6	10 ng/mL	Cell line	Caco-2	E	Increased permeability; decreased occludin and claudin-1 expression; increased claudin-2 level	(119, 120)
IL-22	1 nM	Organoid	Normal colonoid monolayers	E, EE, G, IS	Increased permeability	(121)
IL-22	1-100 ng/mL	Cell line	Caco-2	E	Increased permeability; TJ disassembly	(122)
IL-22	10 and 100 ng/mL	Cell line	T84, HT-29	E	Increased permeability; TJ disassembly	(123)
IL-27	100 ng/mL	Organoid	Normal colonoid monolayers	NR	Attenuated TNFα-induced barrier disruption; increased claudin-4, occludin and E-cadherin expression	(124)
IL-28A	500 ng/mL	Organoid	CD 3-D colonoids	E, EE, G, IS	Increased permeability; decreased expression of E-cadherin and ZO-1	(109)
IL-28A	100 nM	Cell line	Caco-2	E	Decreased permeability; increased expression of claudin-1	(125)
LPS	NR	Organoid	Normal 3-D enteroids	E, G	Increased permeability; decreased expression of ZO-1 and E-cadherin	(126)
LPS	0.3-20 ng/mL	Cell line	Caco-2	E	Increased permeability	(127, 128)

CD, Crohn’s disease; E, enterocytes; EE, enteroendocrine cells; G, Goblet cells; IS, intestinal stem cells; Paneth cells.

NR, not reported.

Primary IEC monolayers derived from human duodenal biopsies developed well-defined TJs and a tight paracellular barrier with TEER ~ 1,000 Ohm x cm^2^, which significantly exceeded the barrier tightness of Caco-2 cell monolayers used as a control (101). Treatment of these enteroid-derived monolayers with different concentrations of TNFα caused a dose-dependent increase in barrier permeability manifested by decreased TEER and increased Lucifer Yellow flux (101). While TNFα triggered IEC apoptosis, it did not enhance transmonolayer passage of a large tracer, 70 kDa dextran, which indicates a lack of significant epithelial cell loss and acellular gaps in the cytokine-exposed monolayers. Immunofluorescence labeling of ZO-1 in enteroid-derived monolayers did not show gross disruption of the TJ integrity by TNFα; however, it did reveal deformation of intercellular junctions manifested by their tortuous morphology (101). Since the increased TJ tortuosity has been previously attributed to the activation of junction-associated NM II (104), it is likely that TNFα disrupted TJ integrity in organoid-derived IEC monolayers by stimulating actomyosin contractility. This suggestion is consistent with another study that examined the effects of TNFα on 3-D small intestinal enteroids (102). TNFα treatment induced global transcriptional alterations in the enteroids. One of the most interesting effects of TNFα was the expressional upregulation of a key NM II activator, myosin light chain kinase (MLCK), although functional consequences of such MLCK induction was not investigated in this report (102). In addition to MLCK upregulation, TNFα had variable effects on TJ proteins by decreasing ZO-2 and ZO-3 expression and increasing mRNA levels for several other junctional proteins, including a ‘leaky’ claudin-2 (102). These data exemplify multiple mechanisms of TNFα-dependent barrier disruption in primary intestinal organoids that involve the altered expression of junctional components and increased contractility of the junction-associated actomyosin cytoskeleton.

Several recent reports investigated the effects of IFNγ treatment of barrier properties of normal human colonoids (99, 108, 109). All of those studies were performed with 3-D spherical colonoids, by measuring FITC-dextran passage into the spheroid’s lumen to evaluate epithelial barrier integrity. While all studies consistently observed the increased dextran permeability in IFNγ-treated colonoids (99, 108, 109), some variability in possible mechanisms of IEC barrier disruption has been reported. For example, IFNγ exposure decreased ZO-1 and occludin expression in primary colonoids, according to one study (99), but did not change expression of these TJ proteins in a similar experimental system in another report (108). These differences may be due to different cytokine concentrations used in the studies, since a higher IFNγ concentration (200 ng/mL) (99) induced more pronounced junctional damage as compared to its lower concentration (50 ng/mL) (108). A combination of TNFα, IFNγ, and IL-1β caused a marked (~ 2 fold) increase in dextran permeability in CD patient-derived colonoids, which was accompanied by alterations in junctional composition (118). Such alterations involved decreased expression claudin-1, angulin-1, E-cadherin, and β-catenin, as well as upregulation of claudin-2 mRNA levels (118). Furthermore, the combined cytokine treatment markedly activated MLCK and STAT1. Importantly, the cytokine-induced barrier leakiness and junctional abnormalities in CD colonoids were reversed by glucocorticoid treatment, thereby highlighting the colonoid model as an attractive *ex vivo* system for testing the barrier protective effects of anti-inflammatory drugs (118).

In addition to TNFα and IFNγ, the effects of few other inflammatory cytokines on assembly and permeability of epithelial junctions in human intestinal organoids have been investigated. Thus, IL-6 treatment was shown to downregulate occludin and claudin-1 mRNA expression in normal colonoids by increasing repressive histone methylation at the promoter regions of these transcripts (119). It is unclear, however, if such transcriptional downregulation resulted in any defects in colonoid permeability or their TJ structure. Another study manufactured an ‘intestinal chip’ based on normal colonoid-derived monolayers and exposed these monolayers on the chip to IL-22, a cytokine relevant to IBD pathogenesis (121). IL-22 treatment disrupted epithelial barrier integrity according to the FITC-dextran flux assay by yet to be defined mechanisms. Similar barrier disruptive effects were attributed to IL-28A, which is a newly-identified member of the interferon family (109). A relatively high concentration of IL-28A (500 ng/mL) increased permeability of 3-D colonoids obtained from CD patients that involved activation of the JAK-STAT signaling pathway and was accompanied by decreased expression of ZO-1 and E-cadherin (109). An interesting example of the barrier protective function has been recently described for IL-27, an immunomodulatory cytokine with anti-inflammatory properties (124). Specifically, exposure of colonoid-derived epithelial monolayers to IL-27 markedly attenuated TNFα-induced barrier disruption and also restored diminished expressions of claudin-4, occludin, and E-cadherin in the monolayers exposed to a combination of TNFα and bacterial lipopolysaccharide (LPS) (124).

While several recent reports describe the effects of inflammatory cytokines on barrier permeability in human intestinal organoids, very little is known about the barrier-disruptive actions of bacterial factors under these experimental conditions. One example is a study aimed at modeling necrotizing enterocolitis by examining responses of 3-D neonatal ileal enteroids to bacterial LPS (126). LPS caused a marked increase in enteroid permeability accompanied by the disruption of both AJs and TJs and diminished mRNA expression of occludin and E-cadherin. By contrast, claudin-2 was significantly upregulated by the LPS treatment (126). Furthermore, LPS stimulated transcription of inflammatory cytokines, TNFα and IL-1β; however, it is unclear if such upregulated cytokine expression was responsible for the described barrier-disruptive effects of LPS.

*Inducible pluripotent stem cell-derived organoids*. Since intestinal organoids generated from human inducible pluripotent stem cells (iPSC) represent more complex structures, retaining both epithelial and mesenchymal compartments, they should more closely recapitulate epithelial responses characteristic of the complex multicellular environment of the intestinal tissue (92–94). Depending on cultured conditions, iPSC-derived intestinal organoids developed either 3-D epithelial structures (103, 110, 129) or 2-D monolayers with well-defined AJs and TJs (114, 115, 130). The epithelial compartment of these organoid was shown to contain all major mucosal cell types, such as enterocytes, Goblet cells, Paneth cells, and enteroendocrine cells, identified by the presence of their lineage-specific protein markers. Interestingly, iPSC differentiated into either colonic or small intestinal monolayers displayed different barrier properties (114). Thus, the TEER values of ~ 1300 Ohm X cm^2^ reported for colon-like monolayers were significantly higher than the TEER of small intestinal-like monolayers (~ 480 Ohm X cm^2^) (114). This is consistent with the known differences in permeability of distinct intestinal segments *in vivo* with the colonic epithelium developing a tighter paracellular barrier as compared to the small intestine (131). Regardless of their morphological appearance (spheres or monolayers), iPSC-derived intestinal organoids responded to inflammatory cytokines with epithelial barrier disruption. Thus, in iPSC-derived intestinal monolayers, exposure to IFNγ and TNFα resulted in decreased TEER and increased transmonolayer FITC-dextran flux (114). Such barrier leakiness was accompanied by the internalization of E-cadherin, disruption of the continuous ZO-1 labeling pattern at TJs, and decreased expression of E-cadherin, ZO-1, and JAM-A (114). Interestingly, in iPSC-derived 3-D intestinal organoids, IFNγ alone did not alter localization of E-cadherin and ZO-1 but caused a selective downregulation of claudin-15 expression (110). By contrast, TNFα treatment of iPSC-derived 3-D organoids resulted in marked disassembly of ZO-1 and occludin-based TJs and increased barrier permeability evident from the enhanced FITC-dextran passage into the spheroids lumen (103).

A unique response of iPSC-derived intestinal organoids to inflammatory stimuli includes the induction of fibrogenic molecular pathways. For example, organoid treatment with TNFα and IL-1α stimulated expression of extracellular matrix proteins, fibronectin and collagen, along with upregulation of a mesenchymal marker α-smooth muscle actin (α-SMA) (132). Furthermore, exposure of iPSC-derived intestinal organoids to a key profibrotic factor, TGF-β, triggered the epithelial-to-mesenchymal transition (EMT) manifested by the increased expression of EMT-related transcriptional factors and the appearance of mesenchymal markers, α-SMA and vimentin, in the epithelial compartment (103, 129).

Overall, the described studies provided solid evidence that the integrity of epithelial barriers developed by either intestinal crypt-derived or iPSC-derived human intestinal organoids become compromised during exposure to key inflammatory cytokines characteristic for IBD mucosa. This highlights intestinal organoids as an attractive model system to evaluate the impact of inflammatory mediators on the intestinal epithelial barrier, investigate their mechanisms of action, and develop novel pharmacological approaches to prevent or reverse compromised epithelial barrier integrity in the inflamed intestinal mucosa.

*Comparison of intestinal organoids with conventional IEC lines*. A large number of studies focusing on the disruption of the gut barrier during mucosal inflammation have been previously performed using conventional IEC lines (17, 32, 33, 36). More recently, these studies are being replicated using human intestinal organoids; hence, there is a need to reconcile the data obtained using new and old experimental systems. It would allow us to answer several important questions. First, do the previous hypotheses and paradigms regarding barrier disruption in the inflamed gut obtained with colon cancer-derived cell lines still hold true, or they need to be replaced with new paradigms? Second, what are the unique responses and mechanisms that could be examined in organoids but not in IEC lines? And finally, could the old and the new experimental systems be used together, or should we abandon the conventional IEC lines and focus exclusively on the primary intestinal organoids?

So far, published studies describe remarkably similar effects of inflammatory stimuli on the barrier properties and junctional integrity in primary human organoids as well as conventional IEC lines. This is particularly evident for the most investigated responses to TNFα and IFNγ. Indeed, earlier studies that exposed Caco-2 and HT-29 cell monolayers with different concentrations of TNFα (10-100 ng/mL) documented the increased barrier permeability (55, 105, 106, 133) and decreased expression of several TJ proteins (105, 107), which resemble the recently-described effects of TNFα in human intestinal organoids (**Table 1**). Furthermore, TNFα similarly increased the expression of the ‘leaky’ claudin-2 in Caco-2 or HT-29 cell monolayers (55, 134) and human enteroids (102), which could contribute to cytokine-induced increase in epithelial permeability. Another common mechanism of TNFα-induced barrier disruption involves increased contractility of the perijunctonal actomyosin ring. This mechanism is supported by the upregulated MLCK expression (102, 106) as well as increased TJ tortuosity (101, 104, 105) observed both in TNFα-treated Caco-2 cells and enteroid-derived cell monolayers.

IFNγ alone or in the combination with TNFα was previously identified as a potent disruptor of epithelial barrier integrity in T84 and Caco-2 cell lines (44, 49, 108, 111–113, 116, 117, 133, 135). This is consistent with the barrier-disrupting effects of this cytokine observed in human intestinal organoids (**Table 1**). Studies addressing the mechanisms of IFNγ-induced disruption of intestinal epithelial barrier yield somewhat controversial results even in similar cellular systems. For example, several reports found decreased expression of the TJ proteins, occludin, and ZO-1, in IFNγ-challenged T84 cells (49, 112, 113), whereas other studies revealed cytokine-induced internalization but not expressional downregulation of these junctional proteins in the same IEC line and using similar IFNγ concentrations (10-100 mg/mL) (44, 108, 111, 136). Likewise, a significant variability of the effects of IFNγ on junctional protein expression was reported by different studies utilizing human intestinal organoids (**Table 1**). A key mechanism responsible for the IFNγ-induced disruption of epithelial junctions in colonic epithelial cell lines involves increased contractility of the actomyosin cytoskeleton that provides driving forces for junctional disassembly. Early studies performed with T84 and Caco-2 cells revealed such increased contractility is driven by activation of the perijunctional NM II motor by its upstream kinases MLCK and Rho-associated kinase (ROCK) (111, 112, 116, 117, 135). Activation of actomyosin contractility was also observed in primary intestinal organoids exposed to the mixture of IFNγ, TNFα and IL-1β, based on a marked upregulation of myosin light chain phosphorylation (118). However, causal roles of MLCK or ROCK-driven activation of perijunctional actomyosin in cytokine-induced barrier disruption in human intestinal organoids have not been yet demonstrated.

In addition to the described actions by TNFα, and IFNγ, other inflammatory mediators appear to have consistent effects on the barrier properties of human intestinal organoids and conventional IEC lines (**Table 1**). For instance, potent barrier-disruptive effects of IL-22 were reported in both Caco-2 monolayers and human colonoids (121–123). Additionally, LPS treatment disrupted TJ barrier in neonatal ileal enteroids and Caco-2 cells (126–128). The only notable example of inconsistent response is the opposite effect of IL-28A in human colonoids and Caco-2 cells (109, 125). Specifically, this cytokine disrupted barrier integrity and decreased AJ/TJ protein expression in colonoids (109) but tightened the paracellular barrier and increased claudin-1 expression in Caco-2 monolayers (125). There are several possible reasons for such contrasting responses. They could be related to different epithelial architectures (3-D spheroids versus Caco-2 monolayers) or the fact that colonoids were derived from CD patients and may display unique, disease-dependent responses. Overall, the described data indicate that the disruptive effects of inflammatory mediators on the epithelial barrier of human primary organoids faithfully reproduced barrier dysfunctions previously described in colon cancer-derived IEC lines. Furthermore, associative evidence suggests that mechanisms underlying epithelial barrier disruption could also be similar in primary intestinal organoids and conventional IEC lines. In this specific field, the utilization of organoids neither resulted in the paradigm shift, nor provided major advances in understanding disruption of the gut barrier during intestinal inflammation.

## Inflammatory mediator induced programmed cell death in intestinal organoids

One of the most obvious benefits of using intestinal organoids is an opportunity to study the roles and mechanisms of the programmed cell death in the inflamed mucosa. Cell death is an important homeostatic process that mediates self-renewal of the normal intestinal epithelium, where terminally-differentiated cells undergo constant apoptosis and shedding from the gut surface into the lumen (137, 138). This homeostatic cell death is known to be dysregulated in the inflamed intestinal epithelium of UC and CD patients (139–141). Active inflammation stimulates several major cell death pathways in IBD mucosa, including apoptosis, necroptosis and pyroptosis (139–141). One could suggest such excessive IEC death would lead to the disruption of the intestinal epithelial barrier by either destabilizing junctional complexes or creating cell-free mucosal wounds. Surprisingly, the causal role of cell death in compromising the IEC barrier remains underappreciated. One reason is studies performed with T84 and Caco-2 epithelial cells concluding that apoptosis does not play a major role in IEC barrier disruption caused by TNFα and IFNγ (44, 116, 133, 142). This conclusion was based on either a lack of apoptotic marker induction in cytokine-treated IEC or the inefficiency of pharmacological inhibition of apoptosis in preventing cytokine-induced barrier breakdown (44, 116, 133, 142). Subsequent studies with murine intestinal organoids, however, convincingly demonstrated TNFα, IFNs, and IL-22 induce strong programmed cell death responses in IEC, primarily *via* the apoptotic and necroptotic mechanisms (143–148). Furthermore, a direct comparison of primary murine organoids and Caco-2 cells exposed to different cytotoxic agents, such as TNFα, chemotherapeutic drugs, and X-ray irradiation demonstrated a high magnitude of cell death in primary organoids and blunted cytotoxic responses of Caco-2 cells (149). This important study provides direct evidence that colon cancer-derived IEC lines could be generally resistant to cell death, presumably due to the activation of pro-survival signaling pathways by their oncogenic mutations.

Several recent studies with human intestinal organoids also suggest that IEC death is a common response to different inflammatory cytokines (**Table 2**). Thus, apoptosis was detected in human duodenal enteroid-monolayers and 3-D colonoids treated with either TNFα alone or in a combination with IL-1β and flagellin (101, 150). Likewise, apoptosis was induced in normal 3-D colonoids and ileal enteroids by a combination of types I, II, and III interferons, and was significantly exaggerated by the co-treatment of interferons and TNFα (152). Another study reported that TNFα induced the necroptotic cell death in small intestinal enteroids derived from CD patients and non-IBD controls by stimulating the expression of several necroptosis mediators including the mixed-lineage kinase domain-like pseudokinase (102). Yet another report suggested the combination of TNFα and IFNγ triggers a non-canonical cell death in colonoids derived from CD patients and non-IBD controls (151). Such non-canonical cell death does not involve apoptotic and necroptotic signaling, but it depends on the JAK1/2-STAT1 activation and non-enzymatic scaffolding activity of caspase 8 (151). In addition to the described cytotoxic effects of TNFα and interferons, other cytokines such as IL-17 and IL-22 were shown to induce different cell death pathways in human primary intestinal organoids (121, 153) (**Table 2**). For example, IL-17A triggered pyroptotic cell death in small intestinal enteroids detected by the cleavage of Gasdermin D and reversed by caspase-1 inhibition (153). On the other hand, IL-22 was shown to induce a classical caspase-3 dependent apoptosis in human colonoid monolayers grown on the intestinal chips (121).

**Table 2:** Inflammatory cytokine-induced cell death in human intestinal organoids.

Cytokine	Cytokine concentration	Type of organoid	Cell lineages present	Type of cell death	Reference
TNFα	25 and 50 ng/mL	Normal enteroid monolayers	E, G, IS	Apoptosis	(101)
TNFα	30 ng/mL	Normal and CD 3-D enteroids	E, G	Necroptosis	(102)
TNFα + IL-1β + Flagellin	100 ng/mL + 10 ng/mL + 100 ng/mL	Normal 3-D colonoids	NR	Apoptosis	(150)
TNFα + IFNγ	10 ng/mL each	Normal and CD 3D colonoids	NR	Non-canonical cell death	(151)
IFNβ + IFNγ + IFNλ	1000 U/mL each	Normal 3-D colonoids and enteroids	NR	Apoptosis	(152)
IL-17A	100 ng/mL	Normal 3-D enteroids	E, EE, G, IS, P	Pyroptosis	(153)
IL-22	1 nM	Normal colonoid monolayers	E, EE, G, IS	Apoptosis	(121)

CD, Crohn’s disease; E, enterocytes; EE, enteroendocrine cells; G, Goblet cells; IS, intestinal stem cells, P, Paneth cells.

NR, not reported.

It should be noted that while the described studies demonstrated the induction of cell death in cytokine-exposed human intestinal organoids, which in some instances was accompanied by increased barrier permeability and the loss of epithelial junctions (101, 102, 121), a causal link between IEC death and barrier disruption is yet to be established. Specifically, there is no data to demonstrate either the pharmacological or genetic inhibition of apoptosis, necroptosis, or pyroptosis could attenuate the barrier breakdown in cytokine-treated organoids. It could be, however, a challenge to prove such a causal relationship, given a recently-proposed concept of the integrated inflammatory cell death program called PANoptosis (154, 155). This concept implies that key programmed cell death pathways could be simultaneously activated by inflammatory stimuli, and they are commonly controlled by a cytoplasmic multiprotein complex, PANoptosome. The individual inhibition of apoptosis, necroptosis, or pyroptosis may not be sufficient to prevent inflammatory cell death that will proceed *via* alternative pathways (154, 155). PANoptosis induction has been recently described in cancer and immune cells treated by the combination of TNFα and IFNγ (156, 157). Such an integrated response could explain the diversity of cell death pathways reported in the cytokine-treated human intestinal organoids (**Table 2**) as well as the inefficiency of pharmacological inhibition of apoptosis in restoring barrier integrity of TNFα/IFNγ treated T84 monolayers (44). We conclude this part by highlighting intestinal organoids as preferred models to study the roles and mechanisms of IEC death in inflamed human intestinal mucosa. Future studies using these experimental systems should shed light on the contribution of the inflammatory cell death pathway in the disruption of the gut barrier in IBD and other inflammatory disorders.

## Compromised barrier integrity and altered composition of apical junctions in intestinal organoids derived from IBD and celiac disease patients

Several recent studies demonstrated that organoids generated from either intestinal crypts or iPSC of IBD and celiac disease patients preserve unique transcriptomic signatures and inflammatory features described for the intestinal epithelium in these diseases (110, 158–162). Such sustained genetic reprogramming has been linked to epigenetic mechanisms, such as changes in DNA methylation patterns (160). One could suggest, therefore, the intestinal organoids also preserve defects in the epithelial barrier integrity and junctional structure characteristics for IBD and other intestinal disorders. Several recent studies tested this idea by investigating the barrier properties and molecular composition of apical junctions in intestinal organoids generated from IBD mucosa (**Table 3**).

**Table 3:** Increased barrier permeability and altered apical junction composition in intestinal organoids of IBD and celiac disease patients.

Type of organoid	Disease type	Cell lineages present	Organoid permeability	Changes in apical junctions	Reference
3-D colonoids	UC	E, G, IS	NR	Increased claudin-2 and claudin-18 expression	(163)
3-D enteroids	CD	NR	NR	Decreased expression of claudins 4, 5 and desmoglein-2; increased claudin-2 expression	(164)
3-D colonoids	CD and UC	E, EE, G	NR	Decreased expression of ZO-1, occludin and claudin-1	(165)
Colonoid monolayers	CD and UC	NR	Decreased TEER	Disruption of tricellular TJs; increased claudin-2 expression	(166)
Colonoid monolayers	CD	NR	Decreased TEER	NR	(167)
Colonoid monolayers	UC	NR	No changes in TEER and dextran flux	NR	(158)
iPSC-derived 3-D organoids	UC	E, G, I, IS, M, T, PR,V	NR	Decreased junctional E-cadherin level	(168)
Enteroid monolayers	Celiac disease	NR	Decreased TEER and increased dextran flux	Decreased claudin-18 and increased claudin-1 expression	(169)

CD, Crohn’s disease; E, enterocytes; EE, enteroendocrine cells, G, Goblet cells, I, immune cells; IS, intestinal stem cells; M, mesenchymal cells; PR, pericytes; T, tuft cells; UC, ulcerative colitis; V, vascular cells.

NR, not reported.

The reported data remain fragmented and contradictory. For example, a study utilizing colonoid-derived epithelial monolayers obtained from actively inflamed tissue biopsies of UC and CD patients reported significant increases in their ionic permeability when compared to the monolayers obtained from non-IBD controls (166). Such barrier leakiness of IBD-derived monolayers was associated with a selective disruption of tricellular TJs and was reversible by pharmacological activation of the AMP-dependent protein kinase (166). Another study reported the increased ionic permeability of colonoid-derived IEC monolayers obtained from non-inflamed CD crypts (167). By contrast, colonic cell monolayers obtained from UC patients had unaltered barrier properties according to TEER and transmonolayer FITC-dextran flux measurements in comparison to the permeability of non-IBD colonoid derived monolayers (158). According to this report, UC colonoids still displayed a diseases-specific transcriptional signature distinct from normal mucosa-derived colonoids, which was not sufficient to cause epithelial barrier disruption (158).

Not only barrier leakiness but also altered molecular composition of epithelial junctions observed in the intestinal mucosa of IBD patients was shown to be preserved in cultured intestinal organoids. For example, a recent study performed a side-by side comparison of the expressional profiles of different junctional proteins in surgically resected ileal samples of CD patients and non-IBD controls to enteroids generated from these tissue samples (164). Importantly, enteroids produced from both severely inflamed and non-inflamed segments of the same tissue samples were examined. This study revealed the decreased protein expression of major TJ, AJ, and desmosomal molecules—such as claudins 1, 4, and 5, E-cadherin, desmoglein-2, and desmocolin-2—both in the non-inflamed and inflamed tissue segments of CD mucosa in comparison to non-IBD controls. By contrast, the expression of claudin-2 protein was upregulated. Interestingly, many of these molecular alterations, including the downregulation of claudins 4 and 5, desmoglein-2, and the upregulation of claudin-2 protein expression were preserved in enteroids generated from the inflamed CD ileum (164). On the other hand, the downregulation of claudin-1, E-cadherin, and desmocolin-2 was not preserved in inflamed CD enteroids. This study also reported an interesting mechanistic observation that the described alterations in junctional composition were at the protein, not mRNA levels. Expectedly, studies examining the expression of junctional proteins in IBD-derived intestinal organoids showed some data variability. Thus, the decreased expression of claudin-1, occludin, and ZO-1 proteins was observed in another cohort of IBD-derived colonoids (165). This contradicts the results obtained with small intestinal enteroids of CD patients, which did not show altered claudin-1 and occludin expression not only in the cultured organoids but even in the original patient tissues (164). Furthermore, while increased expression of claudin-2 protein was consistently found in IBD organoids, such increased protein level was associated with the enhanced claudin-2 mRNA transcription in UC, but not in CD colonoids (163, 164). Possible reasons for such variable results could include differential mucosal responses to inflammation in either of the different IBD types (CD versus UC) or in distinct gut regions (ileum versus colon).

It remains unknown if the altered structure and permeability of epithelial junctions found in IBD intestinal mucosa could be recapitulated in human organoids generated from iPSCs of these patients. Only one study demonstrated concordant decreased expression of E-cadherin along with increased level of RhoA, an important junctional regulator, both in colonic mucosa of UC and paired iPSC-derived intestinal organoids (168). Another study, while demonstrating that iPSC-derived organoids of IBD patients and non-IBD controls respond to proinflammatory cytokines with barrier disruption, did not include sufficient number of samples to detect significant changes in baseline barrier properties of IBD versus non-IBD organoids (114).

Defects in intestinal epithelial barrier integrity also appear to be preserved in enteroids generated from celiac disease patients. Indeed, a recent study that examined duodenal enteroid-derived IEC monolayers reported leakier barrier in celiac disease monolayers as compared to non-celiac controls (169). Such barrier leakiness was manifested by lower TEER and higher transmonolayer FITC-dextran flux. Furthermore, the altered molecular composition of TJs was detected in celiac patient derived IEC monolayers that involved decreased junctional recruitment of ZO-1 and dysregulated expression of several claudin proteins (169). Interestingly, exposure to gliadin, a major trigger of mucosal inflammation in celiac disease, resulted in additional barrier leakiness in celiac, but not control, monolayers that was accompanied by the robust release of several inflammatory cytokines (169). Overall, the described studies identified another valuable application of human intestinal organoids, which provides the unique opportunity to characterize barrier defects of patients with IBD, celiac disease, and other inflammatory disorders as well as understand the molecular mechanisms that underline such barrier dysfunction.

## Conclusion

Human intestinal organoids provide a robust, physiologically-relevant experimental platform to study gastrointestinal homeostasis and diseases. A cadre of recent studies used this novel methodology to model the homeostatic development of the intestinal epithelium and characterize epithelial abnormalities in intestinal disorders (79, 83, 170–172). One of the most attractive applications of intestinal organoid research is to understand the role and mechanisms of intestinal barrier disruption during gut inflammation. There are, however, intrinsic problems and limitations of the organoid utilization, such as their significant heterogeneity, alterations in long-term culture, high data variability, the significant cost and labor intensity of the technology (81, 92, 93, 173). Furthermore, the most frequently used adult stem cell-derived organoids still do not recapitulate the complexity of intestinal tissue due to lack of intraepithelial immune cells and the stromal compartment. Because of these limitations it is important to understand which unique aspect of gut barrier regulation should be investigated using the organoid technology and which could be addressed by working with conventional IEC lines. So far, studies of epithelial barrier disruption in human intestinal organoids recapitulated many phenomena already described in conventional IEC lines and did not discover major novel responses or mechanisms. This does not undermine the value of intestinal organoid research, but, rather, reflects the fact that conventional IEC lines remain valuable models to study intestinal barrier integrity and dynamics under homeostatic conditions and in diseases. There are, however, several particular aspects of intestinal barrier regulation that should be investigated using primary intestinal organoids. Thus, intestinal organoids allow to examine barrier permeability and apical junction integrity in a complex system composed of different intestinal epithelial cell lineages, such as enterocytes, Goblet and Paneth cells. More importantly, primary enteroids represent the only experimental system to study structure and regulation of human small intestinal epithelial barrier due to lack of well-differentiated human small intestinal epithelial cell lines. Likewise, human intestinal organoids represent a superb experimental platform to examine the mechanism of programmed cell death and its effect on intestinal epithelial barrier integrity. Furthermore, patient-derived intestinal organoids provide a unique opportunity to investigate the mechanisms underlying leaky gut barrier in various diseases such as IBD and to develop barrier protecting pharmacological therapies. Finally, iPSC-derived intestinal organoids allow to investigate complex epithelial-stromal interactions and fibrogenic pathways induced by TGF-β and other profibrotic factors in the gut. A particularly important advancement provided by iPSC-derived organoids is the ability to study EMT, junctional disassembly, and tumor metastasis in human colon cancers. In summary, it would be wise to give a negative answer to the question presented in the title of this manuscript. While studying disruption of the gut barrier during mucosal inflammation we should use advances of both traditional IEC cell lines and intestinal organoids in order to understand the roles and mechanisms of this key manifestation of different gastrointestinal disorders.

## Author contributions

Analyzed data and participated in writing, SL, MBN, NN, FR, and AI. Supervision, AI. All authors contributed to the article and approved the submitted version.
